# Analysis of mutations in *pncA* reveals non-overlapping patterns among various lineages of *Mycobacterium tuberculosis*

**DOI:** 10.1038/s41598-018-22883-9

**Published:** 2018-03-15

**Authors:** Ramani Baddam, Narender Kumar, Lothar H. Wieler, Aditya Kumar Lankapalli, Niyaz Ahmed, Sharon J. Peacock, Torsten Semmler

**Affiliations:** 1 Robert Koch Institute, Berlin, 13353 Germany; 2 Department of Clinical Medicine, University of Cambridge, Cambridge, CB2 0QQ United Kingdom; 3 Department of Biotechnology and Bioinformatics, Pathogen Biology Laboratory, School of Life Sciences, University of Hyderabad, Hyderabad, 500084 India; 4 Laboratory Sciences and Services Division, International Centre for Diarrhoeal Disease Research Bangladesh, Dhaka, 1212 Bangladesh; 5 London School of Hygiene and Tropical Medicine, London, WC1E 7HT United Kingdom; 6 Present Address: Laboratory Sciences and Services Division, International Centre for Diarrhoeal Disease Research Bangladesh, Dhaka, Bangladesh; 7 Present Address: Department of Archaeogenetics, Max Planck Institute for the Science of Human History, Jena, Germany

## Abstract

Pyrazinamide (PZA) is an important first-line anti-tuberculosis drug, resistance to which occurs primarily due to mutations in *pncA* (*Rv2043c*) that encodes the pyrazinamidase enzyme responsible for conversion of pro-drug PZA into its active form. Previous studies have reported numerous resistance-conferring mutations distributed across the entire length of *pncA* without any hotspot regions. As different lineages of *Mycobacterium tuberculosis* display a strong geographic association, we sought to understand whether the genetic background influenced the distribution of mutations in *pncA*. We analyzed the whole genome sequence data of 1,480 clinical isolates representing four major *M. tuberculosis* lineages to identify the distribution of mutations in the complete operon (*Rv2044c-pncA-Rv2042c)* and its upstream promoter region. We observed a non-overlapping pattern of mutations among various lineages and identified a lineage 3-specific frame-shift deletion in gene *Rv2044c* upstream of *pncA* that disrupted the stop codon and led to its fusion with *pncA*. This resulted in the addition of a novel domain of unknown function (DUF2784) to the pyrazinamidase enzyme. The variant molecule was computationally modelled and physico-chemical parameters determined to ascertain stability. Although the functional impact of this mutation remains unknown, its lineage specific nature highlights the importance of genetic background and warrants further study.

## Introduction

*Mycobacterium tuberculosis* poses a huge burden on global health, the effective treatment of which is becoming increasingly complex as drug resistance spreads worldwide^1^. According to a WHO report published in 2015 only one in four multidrug resistant-TB cases are diagnosed^2^, highlighting the need for rapid and accurate diagnostic tools. To this end, whole genome sequencing (WGS) offers a better alternative than current methods^3^. In the last few years, many WGS projects have been undertaken around the world to catalogue genetic determinants associated with phenotypic resistance^4–6^. These have largely focused on two key areas. The first is to improve the accuracy in predicting phenotypic resistance based on genetic information, which could then be implemented as an alternative to conventional drug susceptibility testing (DST) and other diagnostic tests. The second is to detect novel mutations associated with drug resistance that are not captured in the current catalogue. Studies have shown the diagnostic potential of WGS to achieve higher diagnostic sensitivity and specificity for some drugs^6–8^, but further work is required to improve the accuracy of prediction for several first and second line drugs that form the core of anti-TB treatment. This includes pyrazinamide (PZA), resistance to which is mainly due to the acquisition of mutations in *pncA* (*Rv2043c*) that impairs the conversion of the pro-drug PZA into its active form pyrazinoic acid^9^. This first-line drug is likely to remain indispensable owing to its bactericidal activity against the persister bacterial sub-population^10^.

Low sensitivity in predicting PZA resistance is largely due to the large number of *pncA* gene mutations observed in phenotypically resistant isolates without clear hotspots for mutations, which poses significant hurdles for the development of genotypic assays^9,11^. Alternative assays such as high-resolution melt analysis (real-time PCR based) that detect variations in the entire *pncA* sequence and upstream promoter region are also limited by the inability to detect genetic events such as transversions or small deletions^12^. Furthermore, phenotypic screening of PZA resistance is challenging since its optimal bactericidal effect requires a low pH, which can lead to inconsistent results^13^.

Previous studies have reported that the propensity to acquire drug resistance varies among different lineages of *M. tuberculosis*^14^. For example, strains belonging to the East Asian lineage (lineage 2) were observed to acquire drug resistance more rapidly than other lineages^15^. Furthermore, strains of different lineages carrying the same resistance mutations have been observed to have different MIC levels for the corresponding drug. These observations clearly highlight the importance of bacterial genetic background^16^. The mechanisms for this lineage specific behavior is surmised to be multifactorial^17,18^, but is poorly understood and requires investigation. It also indicates a need to explore hitherto unknown associations between lineage specific variations and their impact on drug resistance.

Given the above and taking into account the multiplicity of mutations observed in *pncA* gene, we first determined the lineage-wise distribution of resistance conferring mutations identified in a phenotypically PZA resistant strain collection. As *pncA* is part of a larger operon comprising of two additional genes, we also identified the distribution of all mutations in the complete operon and its upstream promoter region using a collection of nearly one and a half thousand *M. tuberculosis* isolates chosen irrespective of their phenotype^6^, mainly to analyze if there exists any lineage specific differences that could impact on PZA susceptibility. This led to the identification of a lineage 3 specific frame-shift deletion in *Rv2044c* gene upstream of *pncA* which resulted in the fusion of these two genes. This added an additional domain of unknown function to the pyrazinamidase enzyme. The variant *pncA* molecule in lineage 3 was computationally modeled and physico-chemical parameters determined and compared to the native molecule. This study emphasizes the importance of analyzing lineage-specific mutations and their potential impact on drug resistance mechanisms in *M. tuberculosis*.

## Results

### Lineage-wise distribution of resistance determining mutations in *pncA*

**Figure. 1:**
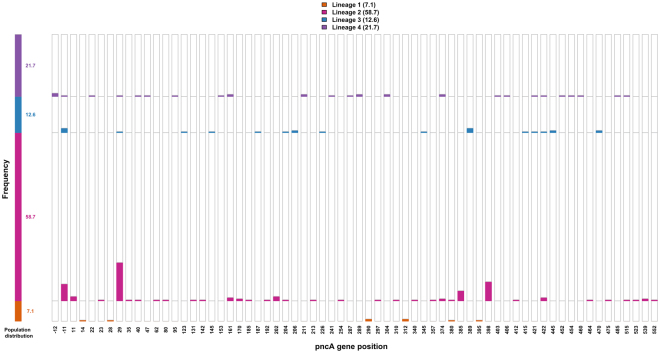
Lineage-wise distribution of resistance determining mutations. The frequency of resistance determining mutations at each distinct position along the gene *pncA* (including upstream promoter region) were analyzed and their lineage information is represented as distinct colored bars. The mutations were spread all across the gene pncA and minimal overlap is observed among the strains of different lineages at any particular positon.

The genome sequence data of 254 phenotypically PZA resistant isolates representing four major lineages - 18 isolates of lineage 1 (7.1%), 149 isolates of lineage 2 (58.7%), 32 isolates of lineage 3 (12.6%) and 55 isolates of lineage 4 (21.7%) - were analyzed to identify the resistance determining mutations in gene *pncA* (including 40 bp upstream region). Out of 254 strains investigated, resistance determining mutations were observed in 181 isolates (71.2%) comprising 8 isolates of lineage 1, 118 isolates of lineage 2, 24 isolates of lineage 3 and 31 isolates of lineage 4. Complete information for these mutations is provided in Supplementary Table S1. The lineage-wise distribution of resistance determining mutations and their frequency at each distinct position along the gene are shown in Fig. 1. This clearly showed an absence of overlap among lineages at 85% of the positions where mutations were identified, for example as shown at positions 202 and 385. Mutations that confer resistance in *M. tuberculosis* are generally acquired via convergent evolution in different lineages, an example being mutations in *rpoB* that confers resistance to rifampicin and are concentrated in the rifampicin resistance determining region^19^ as shown in Supplementary Figure S1 (complete details available in Supplementary Information). By contrast, here we observed that resistance determining mutations were distributed across *pncA* with minimal overlap observed between strains of different lineages at any particular position.

### Lineage-wise distribution of mutations in the complete operon (*Rv2044c-pncA-Rv2042c*)

Genome sequence data for 1480 *M. tuberculosis* isolates belonging to the major lineages were selected irrespective of phenotype from publically accessible genomes that were generated by a previous study^6^. The process for selecting isolates is described in the methodology. Our collection represented all four major lineages - 141 isolates of lineage 1 (9.5%), 91 isolates of lineage 2 (6.1%), 315 isolates of lineage 3 (21. 3%) and 933 isolates of lineage 4 (63%). It was previously reported that *pncA* (*Rv2043c*) is co-transcribed as a polycistron along with *Rv2044c* located 40 base pairs (bp) upstream to *pncA*, and *Rv2042c* is located immediately downstream with a 1 bp overlap with *pncA*^20^. To capture this entire region, 1801bp sequence of H37Rv reference strain (NC_000962.3) corresponding to the complete operon (*Rv2044c-pncA-Rv2042c*) together with 85 bp upstream (to include the promoter region for the operon) was used for variant calling with GATK HaplotypeCaller. After filtering for variants using metrics described in the methodology, mutations were detected at 68 distinct positions in the operon. The lineage-wise distribution of all the single nucleotide polymorphisms (SNP) and insertion/deletion (indels) sites is shown in Table 1. Complete details of genetic variants at each position and their frequency are provided in Supplementary Table S2.

**Table 1:** Comparative statistics of single nucleotide polymorphism (SNP) and insertion/deletion (indels) sites observed among different lineages.

	No. of indel sites	No. of SNP sites
Lineage 1	2	9
Lineage 2	0	11
Lineage 3	5	10
Lineage 4	4	29

**Figure 2:**
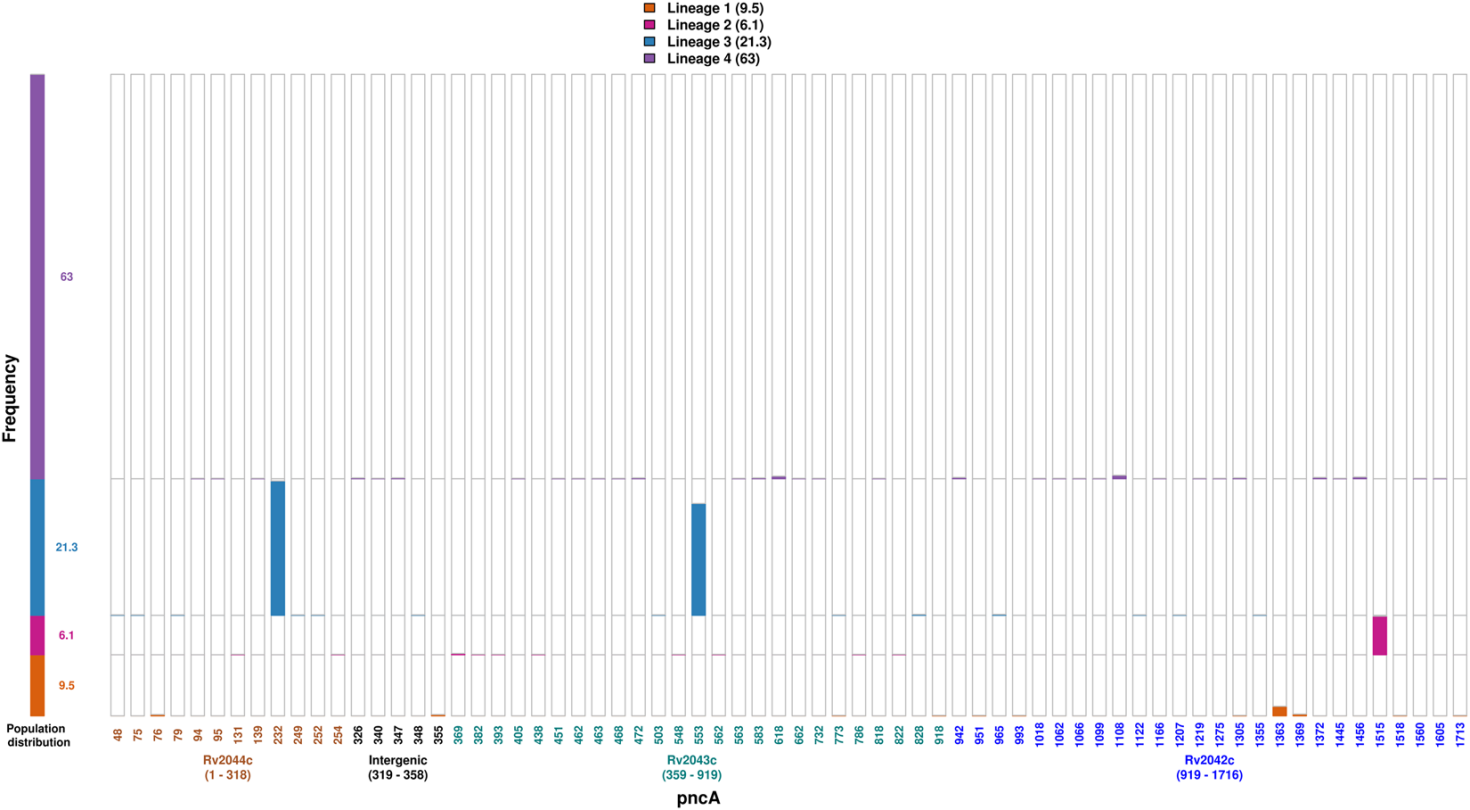
Lineage-wise distribution of mutations in the complete operon (*Rv2044c-pncA-Rv2042c*). The frequency of mutations at each distinct position along the operon (-85bp -*Rv2044c*-*Rv2043c(pncA)*-*Rv2042c*) were determined and their lineage information represented above. The position of mutation corresponds to location in the operon and different colors represent genes in which they are identified – *Rv2044c* (brown), intergenic region (black), *Rv2043c* (green), *Rv2042c* (blue). The two genetic variants showed lineage specific behavior being present in 97% of isolates belonging to that lineage and completely absent in others, as follows - single nucleotide deletion (GCCG/232/GCG) in *Rv2044c* observed in isolates of lineage 3 and SNP (C1515G) in *Rv2042c* observed in isolates of lineage 2.

Figure 2 illustrates the position and frequency of the genetic variant alleles at each of these 68 distinct positions along the operon. Variant positions rarely overlapped in different lineages, with two exceptions. The first was present in *pncA* where a single nucleotide deletion (GT/773/G) was identified in lineage 1 strains and a SNP (G/773/C) at the same locus in lineage 3 strains. The other variant position was present in *Rv2042c* where lineage 1 strains carried a SNP (G/1305/A) whereas lineage 4 strains harbored SNP (G/1305/C) at the same locus (Supplementary Table S2).

Our analysis also identified variants at two positions that were highly specific to strains of a particular lineage (present in 97% of isolates belonging to that lineage and absent in the remaining isolates/lineages) as shown in Fig. 2. The first was a SNP (C1515G) in *Rv2042c* in lineage 2 isolates, and the second was a single nucleotide deletion (GCCG/232/GCG) in *Rv2044c* in lineage 3 isolates. Apart from the above mentioned two variants, SNP (C553T) in *pncA* was observed in 81% of lineage 3 isolates. This has been reported previously as a lineage 3-associated silent mutation (Ser65Ser)^19^, which was absent in a small number of basal isolates of this lineage.

### Effect of lineage-specific variations

**Figure 3:**
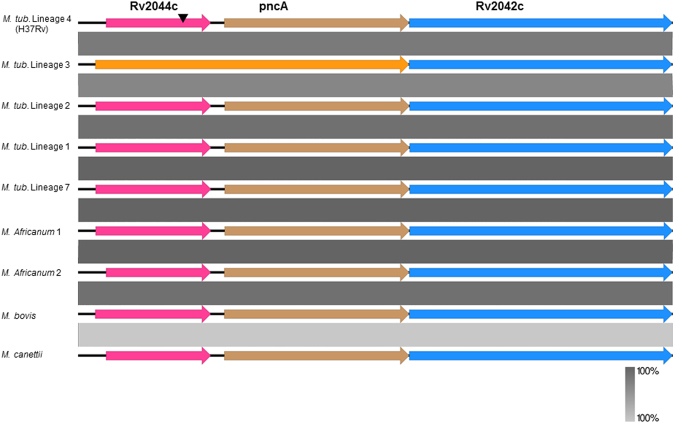
Comparison of (*Rv2044c-pncA-Rv2042c)* in MTBC and *M*. canettii. Comparison of genes *Rv2044c* (pink), pncA (brown) and *Rv2042c* (blue) among different lineages of *Mycobacterium tuberculosis* complex and *Mycobacterium canettii* reveal a high degree of conservation. A frameshift deletion (arrow in black) in *Rv2044c* resulted in its fusion with pncA gene which was restricted to isolates of Lineage 3 (variant in orange). Conserved regions are represented using the gradient scale in grey.

The lineage 2 specific SNP (C/1515/G) in *Rv2042c* was a synonymous substitution with no change in amino acid sequence. By contrast, the deletion of a single nucleotide (GCCG/GCG) at position 232 in *Rv2044c* caused a frameshift that disrupted the stop codon and resulted in fusion of *Rv2044c* with the downstream *pncA* to create a hybrid molecule consisting of 305 amino acids only in lineage 3 of *M. tuberculosis* as shown in Fig. 3. In silico analysis of this hybrid molecule using NCBI CD-search^21^ indicated the addition of a new domain of unknown function (DUF2784) previously encoded by *Rv2044c* to the variant *pncA* of lineage 3. The DUF2784 domain is reported to be a conserved domain in bacteria, but has not been functionally characterized. This observation led us to analyze the status of these genes (*Rv2044c-pncA-Rv2042c*) in other lineages of MTBC, non-tuberculous mycobacteria (NTM) and *M*. *canettii* to evaluate its evolutionary significance.

### Comparison of (*Rv2044c-pncA-Rv2042c)* in MTBC and *M*. canettii

*M. tuberculosis* belongs to the *Mycobacterium tuberculosis* complex (MTBC), which contains other human and animal adapted lineages including *M. africanum* and *M. bovis*, respectively^22^. Different MTBC lineages have evolved clonally over time after their divergence from the common ancestor with smooth tubercle bacilli, *M*. *canettii*^23^. A NCBI BLASTn^24^ comparison was carried out to determine the orthologs of genes *Rv2044c-pncA-Rv2042c* as well as to identify whether the deletion in *Rv2044c* was present in other members of the MTBC and *M*. *canettii*. As shown in Fig. 3 (a comparison made using EasyFig^25^), these genes are highly conserved among various lineages of MTBC and *M*. *canettii*, but the lineage 3 specific frameshift deletion in *Rv2044c* was not identified in representative isolates of all other lineages considered, hinting that the deletion may have occurred post lineage diversification in *M. tuberculosis*. The status of these genes in the majority of NTM isolates has been already reported in a previous study^26^.

### Modeling of variant pyrazinamidase enzyme of *M. tuberculosis* lineage 3

Physicochemical properties of the variant PncA molecule were compared to the native PncA protein using ProtParam, a summary from which is shown in Table 2. The isoelectric point of the variant PncA protein indicates that the variant protein is more basic compared to the native protein. An increase in the number of positively charged residues was also observed. An instability index of less than 40 for both proteins along with similar aliphatic indices indicates that both proteins are stable. An increase in GRAVY score indicates that the variant PncA protein is more hydrophobic compared with the native protein.

**Table 2:** *In silico* comparison of physico-chemical properties of original PncA molecule with that of variant one observed only in lineage 3.

Protein	pncA (native)	pncA (L3 lineage)
No. of amino acids	186	305
Molecular weight	19604.64	32931.31
Theoretical pI	4.43	6.25
No. of negatively charged residues (Asp + Glu)	27	30
No. of positively charged residues (Arg + Lys)	10	26
Instability index	10.35	29.97
Aliphatic index	83.92	83.51
Grand average of hydropathicity (GRAVY)	0.002	0.055

**Figure 4:**
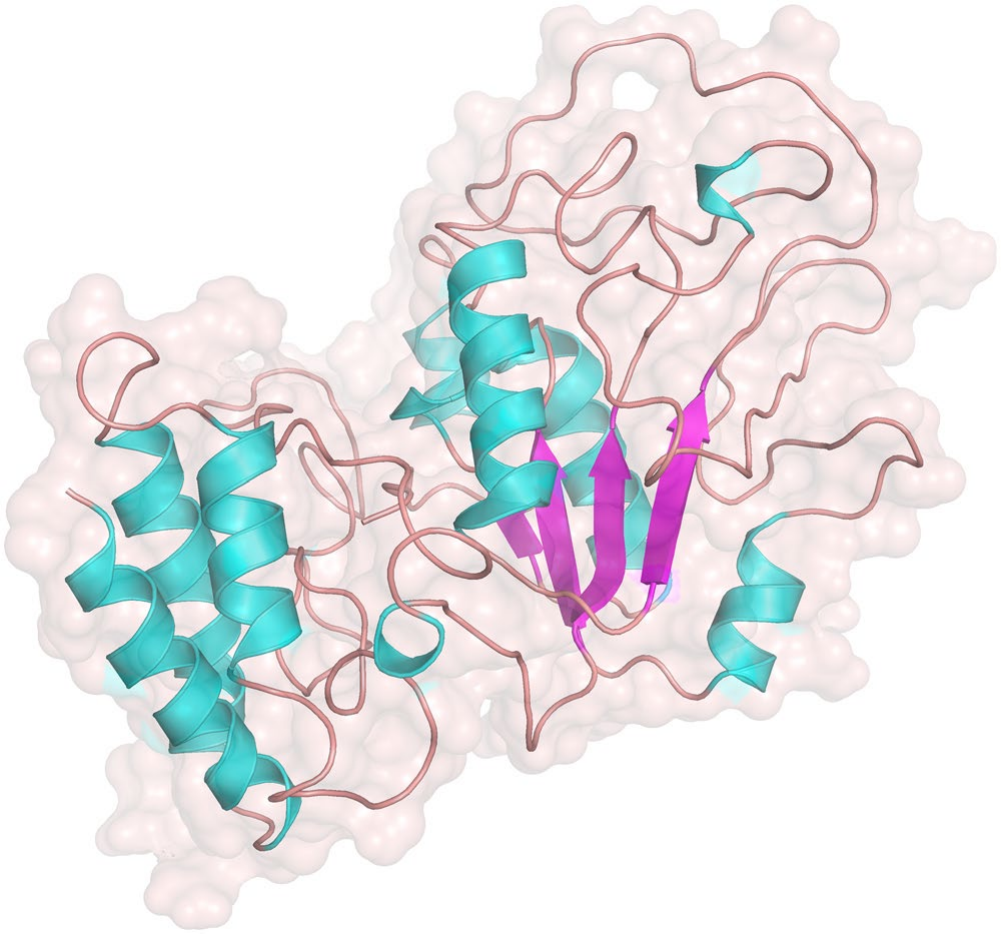
3D structure of the variant pyrazinamidase molecule. The 3D structure was visualised using Pymol - Helix (cyan), sheets (magenta) and coil(brown).

**Figure 5:**
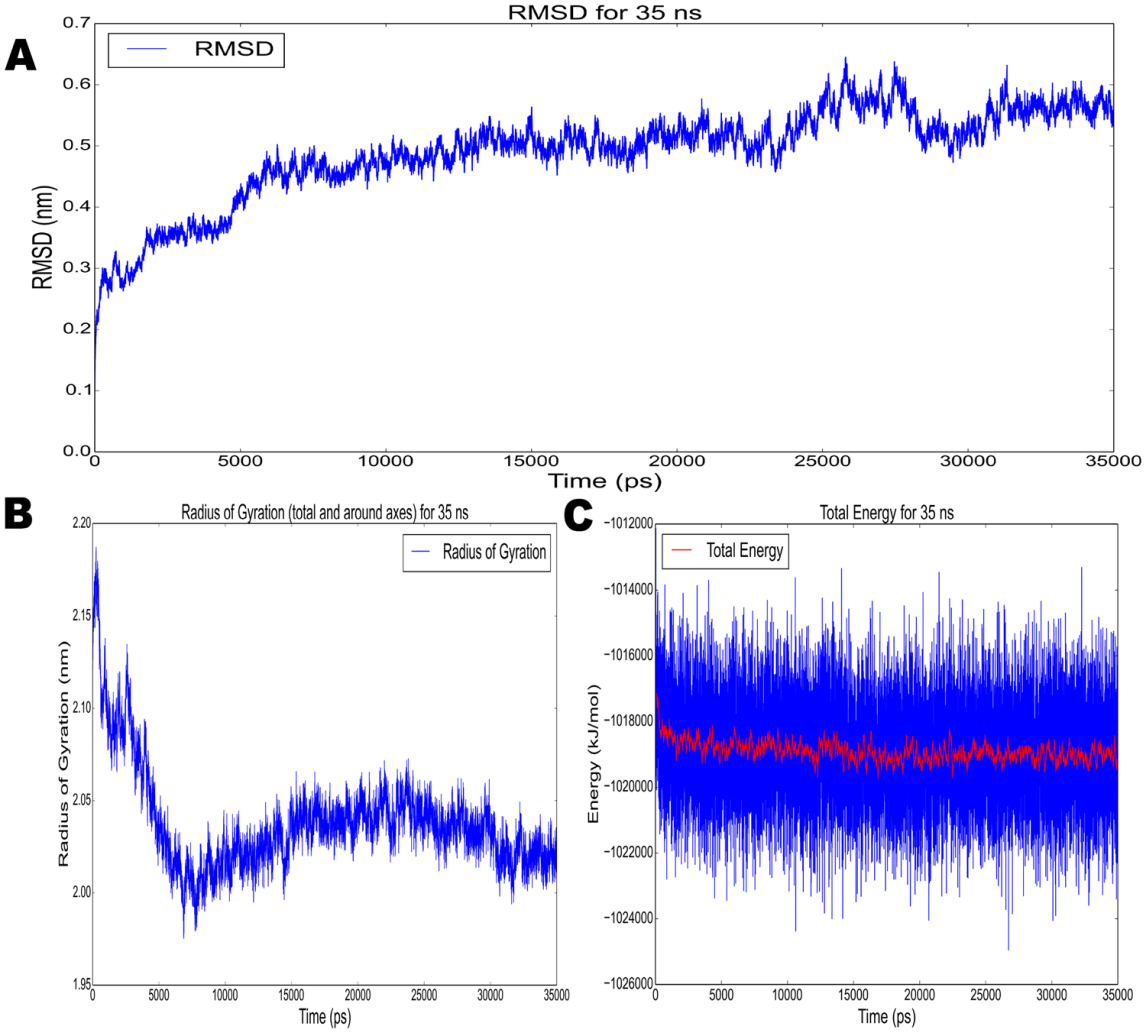
Model refinement analysis using gromacs - (**A**) RMSD, (**B**) Radius of gyration, (**C**) Total Energy. The flattening of the RMSD plot of the protein backbone was observed around 35 ns as shown in (**A**). The plot of gyration radius was also stable around 2.4 A° representing the compactness of the protein during the simulation as shown in (**B**). The total energy trajectory remained stable over the entire simulation period at around −1.018e + 3 KJ/mol, as shown in (**C**).

The crystal structure of native pyrazinamidase has been determined by Petrella *et al*.^27^, and was available in the RSCB Protein Databank (PDB). An *in silico* evaluation was performed to determine a representative conformation of the variant pyrazinamidase enzyme in equilibrium. 3D modelling was performed using I-TASSER software, and refinement of the protein 3D model was performed in multiple steps to address the lack of homology to the extended region in the variant enzyme (see methodology). The final model with the highest confidence score contained 31.4% Alpha helix, 9.5% Strand, 2.9% 3–10 helix, and the remainder included mainly Coil, as shown in Fig. 4. The Ramachandran plot assessment of residues of the 3D model using RAMPAGE revealed 87.8% residues in favorable region, 7.3% in allowed region, and 5% comprised outliers. This final model was further subjected to MD simulations for 35 ns using Gromacs and the stability and conformational changes were analyzed. The flattening of the RMSD plot of the protein backbone around 35 ns in Fig. 5A indicated that the molecule achieved a stationary phase during the later stages of simulation and showed fluctuations around 5.4 A° at the end of simulations. The plot of gyration radius was also stable around 2.4 A° with no major modifications in the secondary structure of the protein, representing the compactness of the protein during the simulation as shown in Fig. 5B. The total energy trajectory remained stable over the entire simulation period at around −1.018e + 3 KJ/mol, as shown in Fig. 5C. Ramachandran plot analysis of the model obtained after MD simulation depicted 86.8% residues in favorable region, 10.3% in allowed region and 3.0% in disallowed regions.

## Discussion

*M. tuberculosis* has co-evolved with humans over time, which has led to the emergence of different geographically compartmentalized bacterial lineages^28^. Genetic diversity of these lineages may influence both virulence and transmission potential^29^. In addition, strains from specific lineages have been shown to have an increased capacity to acquire resistance or mitigate the associated fitness cost^30^. This supports the importance of a detailed understanding of the effect of genetic background on biological traits, including drug resistance. The genetic variation among lineages is also pertinent in relation to pyrazinamide where resistance conferring mutations are diverse and distributed across *pncA*. Hence, we analyzed the lineage wise distribution of mutations in the operon comprised of *Rv2044c*, *pncA* and *Rv2042c*.

We identified an important frameshift deletion in *Rv2044c* affecting *pncA* that was restricted to *M. tuberculosis* lineage 3. This frameshift deletion disrupted the stop codon of *Rv2044c*, and the hybrid molecule composed of the two genes effectively increased length of PncA by 119 amino acids. As *pncA* is reported to be expressed as part of a polycistronic mRNA product of the operon^20^, any effect on expression due to this deletion would be minimal. This observation is supported by the evidence of enzymatic activity in lineage 3 isolates harboring this deletion, as reported recently by Miotto *et al*.^31^. In their study on pyrazinamide resistance, sequencing of *pncA* was carried out for 1950 clinical isolates and upstream promoter region (>100 bp) was also included for a small number of isolates.

This strongly suggests that the lineage-specific variation identified in this study does not disrupt pyrazinamidase enzyme activity and that a variant enzyme with an additional domain DUF2784 of unknown function is transcribed in isolates of lineage 3. Furthermore, structural refinement of the 3D model obtained from I-TASSER using MD simulations showed that the variant molecule is stable as displayed by the trajectories of RMSD, total energy and radius of gyration for 35 ns, which validates the conformation of the predicted structure. Minor fluctuations observed in the trajectories could be attributed to the presence of coil regions in the protein model, which are difficult to stabilize during the simulations. However, functional studies comparing the native and variant PncA are necessary to elucidate the role of the additional domain and its full impact on enzyme function.

To evolve from an environmental bacterium into a human restricted professional pathogen, *M. tuberculosis* could have selected many of the attributes that are found commonly in non-tuberculous mycobacteria (NTM) and smooth tubercle bacilli (*M. canettii*). A previous study that compared genomes of MTBC with 11 other related NTM species comprising both free living environmental bacteria and opportunistic pathogens^26^ revealed that orthologs of *Rv2042c* and *pncA* were conserved in the majority of NTM except for *M*. *leprae*, which has undergone extensive genome reduction^32^. The third member of the operon (*Rv2044c* ortholog) was absent in the majority of NTMs including slow growing species such as *M*. marinum, *M. leprae* and *M*. avium, the exception being *M*. smegmatis. The evolution of different slow growing mycobacterial species from their common ancestor involved the acquisition or deletion of various important genes that are species-specific or shared by small number of slow growing mycobacteria^33,34^. The presence of orthologs of *Rv20444c* in a limited number of slow growing mycobacterial species and all MTBC members indicates its importance in the latter. Although all members of the MTBC harbor an ortholog of *Rv2044c*, the evolutionary fixation of the frameshift deletion in lineage 3 isolates identified here strongly suggests that the variant *pncA* represents a beneficial adaptive change in the associated geographical setting.

A recent multi-country surveillance report which assessed levels of pyrazinamide and rifampicin resistance in five different high burden countries observed that levels of pyrazinamide resistance were significantly lower compared to the levels of rifampicin resistance only in Pakistan^35^. Although this study did not report lineage information, other reports have shown the predominance of lineage 3 in Pakistan and Northern India (Delhi)^36–38^. This raises the possibility of alternative mechanisms of pyrazinamidase enzyme action in these strains. Therefore, mechanisms by which *M. tuberculosis* lineage 3 strains acquire PZA resistance need careful investigation.

The genetic background constituted by lineage specific non-resistance conferring mutations might influence drug resistance and amelioration of their impact through epistatic interactions^17^. To our knowledge, our study is the first to report the effect of a lineage-specific non-resistance mutation on the drug resistance associated gene, *pncA*. Moreover, this study highlights the need for examination of lineage-specific variations, mainly indels whose downstream effect is commonly not estimated compared to SNPs. Minimal overlap in the position of variants between lineages could be affected by the limited number of isolates tested for some lineages. Future studies of large bacterial collections containing under-sampled lineages promise to yield better resolution and provide deeper insights into the dynamics of resistance acquisition.

## Methods

### Screening for mutations

Intitially, the read data of 254 strains reported as phenotypically resistant to PZA were considered from two previous studies^6,37^ in order to identify the resistance determining mutations along the gene *pncA*. The sequence reads were aligned to the region of H37Rv corresponding to *pncA* gene including 40 bp upstream region. Further, to identify all the genetic variants along the operon, out of 3651 genome sequences made available by a previous study from Walker *et al*.^6^, the read data that fulfilled the following criteria were chosen randomly irrespective of phenotype - read length of 75 bp,100 bp and 101 bp with coverage greater than or equal to 100× (complete accession details are provided in Supplementary Table S3). These were downloaded from the NCBI sequence read archive using the SRA tool kit^39^. The sequence reads belonging to different lineages of *M. tuberculosis* were aligned to the region of H37Rv corresponding to (*Rv2044c-pncA-Rv2042c*) genes and 85 bp upstream to identify variants.

### Variant calling and annotation

This variant calling step involved generation of an alignment file using bwa-mem^40^ and further processed using Picard tools^41^ to mark duplicate reads before applying the GATK HaplotypeCaller^42^. All variations called in the VCF (variant call format) file were filtered for metrics – QUAL (>50), DP (>10), MQ (>40), QD (>20). Further, filtering based on AD values was performed to include only those sites where reads supporting alternate allele were greater than or equal to 75 percent of total reads aligned at that position. The annotation of variants and identification of resistance determining ones was done using in-house written python scripts. The effect of lineage specific mutations were visualized using Expasy translate tool^43^ and confirmed with gene prediction software GeneMarkS^44^.

### Domain identification and structure analysis of variant protein

The prediction of domains was done using NCBI CD-search^21^. Physico-chemical properties (molecular weight, theoretical PI, instability index^45^, aliphatic index^46^, GRAVY score^47^) were determined for the native and variant proteins using Prot-Param server^48^. The three-dimensional (3D) model of the variant protein comprising 305 amino acids (aa) was initially predicted by ab-inito modeling in I-TASSER online server^49^. The top 10 threading templates detected by the I-TASSER included a template file corresponding to the original pyrazinamidase crystal structure (3PL1-186 aa) PDB as the top most alignment, but also none of the other templates had any alignment to the newly added region of the protein. In order to overcome this effect, the first 119 aa sequence which did not have any reported alignment was modeled ab-initio separately again using I-TASSER server. In the last step, the model obtained in previous run for first part of the protein was provided as a user template while submitting complete sequence to I-TASSER for final 3D modeling.

The best model obtained in final run was chosen based on C-score and visualized in PyMOL^50^ before subjecting to structure refinement and validation analysis. Molecular Dynamics (MD) simulations were performed using GROMACS version 5.1.4^51^. This mainly included topology generation with GROMOS96 54a7 force field in an aqueous environment, solvation of defined cubic box, neutralization of charge using 4 sodium ions and geometry optimization. Initial unconstrained global dynamics was carried out in two steps - temperature (300 K) for 100 picoseconds followed by Pressure (1 bar) for 100 picoseconds. The final step of MD simulations for 35 nanoseconds was performed at pressure (1 bar) and temperature (300 K).The Ramachandran plots of variant pyrazinamidase before MD simulation and final one obtained after MD simulation were compared using RAMPAGE tool^52^.

### Data Availbility Statement

All the read data analysed is availble on NCBI and accession details are provided.

## Electronic supplementary material

Supplementary Information

Supplementary Table S1(XLS 112 kb)

Supplementary Table S2(XLS 25 kb)

Supplementary Table S3
