# Author Correction: Analysis of mutations in *pncA* reveals non-overlapping patterns among various lineages of *Mycobacterium tuberculosis*

**DOI:** 10.1038/s41598-018-25809-7

**Published:** 2018-05-15

**Authors:** Ramani Baddam, Narender Kumar, Lothar H. Wieler, Aditya Kumar Lankapalli, Niyaz Ahmed, Sharon J. Peacock, Torsten Semmler

**Affiliations:** 1 Robert Koch Institute, Berlin, 13353 Germany; 2 Department of Clinical Medicine, University of Cambridge, Cambridge, CB2 0QQ United Kingdom; 3 Department of Biotechnology and Bioinformatics, Pathogen Biology Laboratory, School of Life Sciences, University of Hyderabad, Hyderabad, 500084 India; 4 Laboratory Sciences and Services Division, International Centre for Diarrhoeal Disease Research Bangladesh, Dhaka, 1212 Bangladesh; 5 London School of Hygiene and Tropical Medicine, London, WC1E 7HT United Kingdom; 6 Present Address: Laboratory Sciences and Services Division, International Centre for Diarrhoeal Disease Research Bangladesh, Dhaka, Bangladesh; 7 Present Address: Department of Archaeogenetics, Max Planck Institute for the Science of Human History, Jena, Germany

Correction to: *Scientific Reports* 10.1038/s41598-018-22883-9, published online 15 March 2018

The Acknowledgements section in this Article is incomplete.

“We thank Dr. Astrid Lewin for her helpful discussion concerning the mechanism of PZA action. This publication presents independent research supported by the Health Innovation Challenge Fund (WT098600, HICF-T5-342), a parallel funding partnership between the Department of Health and Wellcome Trust. The views expressed in this publication are those of the author(s) and not necessarily those of the Department of Health or Wellcome Trust.”

should read:

“We thank Dr. Astrid Lewin for her helpful discussion concerning the mechanism of PZA action. This publication presents independent research supported by the MRC-DBT funded partnership between National Institute for Research in Tuberculosis, Chennai and University of Cambridge (MR/N501864/1, NK). The views expressed in this publication are those of the author(s) and not necessarily those of the MRC or DBT.”

