# Supplementary material for: Analysis of mutations in pncA reveals non-overlapping patterns among various lineages of Mycobacterium tuberculosis
Source: Sci Rep. 2018 Mar 15;8:4628. doi: 10.1038/s41598-018-22883-9 (PMC5854631; doi:10.1038/s41598-018-22883-9)
Supplement: Supplementary file 1 — Supplementary Information [file 41598_2018_22883_MOESM1_ESM.doc]

**Analysis of mutations in *pncA* reveals non-overlapping patterns among various lineages of *Mycobacterium* tuberculosis**

Ramani Baddam^1,#a^, Narender Kumar^2^, Lothar H. Wieler^1^, Aditya Kumar Lankapalli^3,#b^, Niyaz Ahmed^3,4^, Sharon J. Peacock^2,5^, Torsten Semmler^1*^

^1^Robert Koch Institute, Berlin, 13353, Germany

^2^Department of Clinical Medicine, University of Cambridge, Cambridge, CB2 0QQ, United Kingdom

^3^Pathogen Biology Laboratory, Department of Biotechnology and Bioinformatics, School of Life Sciences, University of Hyderabad, Hyderabad, 500084, India.

^4^Laboratory Sciences and Services Division, International Centre for Diarrhoeal Disease Research Bangladesh, Dhaka, 1212, Bangladesh.

^5^London School of Hygiene and Tropical Medicine, London, WC1E 7HT, United Kingdom

^#a^Present address: Laboratory Sciences and Services Division, International Centre for Diarrhoeal Disease Research Bangladesh, Dhaka, Bangladesh

^#b^ Present address: Department of Archaeogenetics, Max Planck Institute for the Science of Human History, Jena, Germany

**Supplementary Material**

**Supplementary Figure S1: Lineage wise distribution of resistance determining mutations of Rifampicin**

The frequency of resistance determining mutations at each distinct position along the gene *rpoB* were analyzed and their lineage information is represented as distinct colored bars. The mutations were mainly focused in the rifampicin resistance determining region and different lineages were observed to be share certain positions displaying a convergent acquisition.

**Supplementary Table S1:** Details of pyrazinamide and rifampicin resistance determining mutations.

**Supplementary Table S2:** Genetic variants detected at each position in the operon and their frequency in 1480 *M. tuberculosis* isolates belonging to different lineages.

**Supplementary Table S3: Accession details of read data**

The read data accession details for all 1480 isolates (sheet 1) and for pyrazinamide and rifampicin resistant isolates (sheets 2 & 3, respectively).

**Supplementary Figure S1**

**
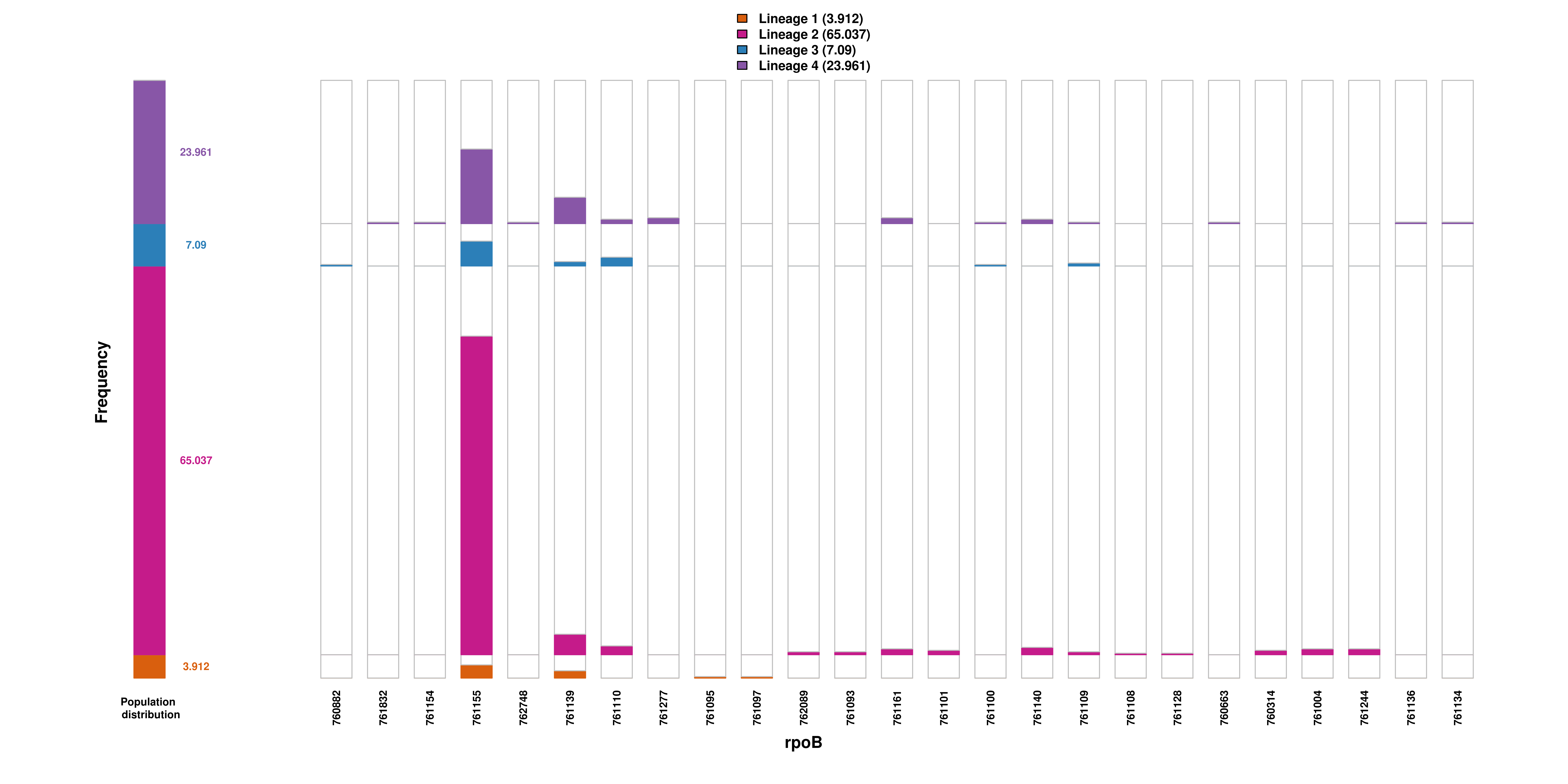
**

**Supplementary Methods**

**Determination of resistance determining mutations of rifampicin**

In order to visualize the lineage wise distribution of resistance determining mutations and rifampicin, the read data of strains which were listed as phenotypically resistant to rifampicin were identified separately from the study of Walker et al [1] and downloaded using NCBI SRA tool kit. The Rifampicin resistant collection identified included 16 isolates of lineage 1(3.9%), 266 isolates of lineage 2 (65%), 29 isolates of lineage 3(7%) and 98 isolates of lineage 4 (23.9). The complete accession details of rifampicin resistant isolates are provided in Supplementary Table S3.

The sequence reads belonging to different lineages of *M.* *tuberculosis* were aligned to the region of H37Rv corresponding to *rpoB* for identifying variants. The variant calling protocol and filtering metrics used here are same as described in the methodology section. The high quality variants obtained after filtering were annotated and analyzed using custom made python scripts. The genetic variants which are listed in the previous literature as resistance determining mutations were only considered for final plotting. The complete details of these resistance determining mutations along with their reference information is provided in Supplementary Table S1.

**References**

1. Walker TM, Kohl TA, Omar S V., Hedge J, Del Ojo Elias C, Bradley P, et al. Whole-genome sequencing for prediction of Mycobacterium tuberculosis drug susceptibility and resistance: A retrospective cohort study. Lancet Infect Dis. 2015;15: 1193–1202. doi:10.1016/S1473-3099(15)00062-6
